# In Vivo CAR-Based Immune Cell Engineering: Future Applications and Challenges in Malignant Glioma

**DOI:** 10.3390/cancers18121986

**Published:** 2026-06-18

**Authors:** Junya Yamaguchi, Alejandra Bergquist, Jianwen Lu, Senthilnath Lakshmanachetty, Safwaan H. Khan, Hideho Okada

**Affiliations:** 1 Department of Neurological Surgery, University of California, San Francisco, CA 94158, USA; 2 Parker Institute for Cancer Immunotherapy, San Francisco, CA 94129, USA

**Keywords:** in vivo engineering, CAR-T, malignant glioma, glioblastoma

## Abstract

Chimeric antigen receptor (CAR)-T cell therapy has achieved remarkable success in hematologic malignancies, and its development is being actively pursued across a broad range of cancer types. However, current CAR-T cell therapies rely on ex vivo engineering, which presents significant logistical, temporal, and biological limitations. In vivo CAR-T cell engineering is emerging as a new paradigm that may overcome these challenges by enabling the direct reprogramming of immune cells within the patient through the administration of CAR-encoding vectors. This approach represents an off-the-shelf form of autologous immune therapy. Advances in viral engineering and nanotechnology have enabled the development of diverse CAR delivery platforms that not only deliver CAR constructs but also facilitate the delivery of gene-editing components, such as Cas9, allowing for more sophisticated in vivo genetic modifications. Some of these approaches have already entered clinical evaluation and have shown promising early results in hematologic malignancies, with clinical trials in solid tumors now underway. However, the application of in vivo-engineered CAR-T cell therapies to malignant glioma remains largely unexplored, reflecting challenges distinct from those encountered in hematologic malignancies. In this review, we discuss these challenges and potential strategies to address them, while highlighting recent progress in in vivo CAR-T cell engineering.

## Simple Summary

Chimeric antigen receptor (CAR)-T cell therapy has shown strong success in blood cancers, but its current manufacturing process is complex, time-consuming, and difficult to scale. A new approach, called in vivo CAR-T cell engineering, aims to overcome these limitations by directly programming a patient’s immune cells inside the body using specially designed delivery systems. This strategy could simplify treatment and make it more widely accessible. Recent advances in viral and nanoparticle technologies have enabled the development of platforms that not only deliver CAR constructs but also support more precise genetic modifications. While early clinical studies in blood cancers have shown promising results, applying this approach to malignant glioma remains challenging due to unique biological barriers. This review highlights current progress in in vivo CAR-T cell engineering and discusses key obstacles and potential strategies for improving its application to malignant glioma.

## 1. Introduction

Chimeric antigen receptors (CARs) are synthetic fusion proteins composed of an antigen-recognition domain, typically derived from a single-chain variable fragment (scFv), linked to intracellular signaling domains, including costimulatory molecules and CD3ζ. CAR-T cell therapy, in which T cells are genetically engineered to express CARs, has achieved remarkable clinical success in treating hematological malignancies. However, despite substantial progress in improving CAR design from structural, functional, and logistical perspectives, its efficacy against solid tumors remains limited.

Currently, the standard manufacturing approach for CAR-T cells relies on ex vivo engineering of autologous T cells. However, this manufacturing process is labor-intensive, time-consuming, and costly, severely limiting scalability and accessibility. These challenges have driven the development of off-the-shelf CAR-T cell products.

In vivo CAR-T cell engineering—where CAR-T cells are generated directly within the patient—has gained considerable attention as a promising approach to overcome the limitations of conventional ex vivo manufacturing. Advances in viral vector engineering and nanoparticle-based delivery systems have enabled the clinical translation of in vivo gene therapies for diseases, such as neurodegenerative disorders and hemophilia, paving the way for similar strategies in cancer immunotherapy [1,2,3]. In vivo CAR-T cell engineering not only offers economic and logistical advantages but is also expected to provide biological benefits, including improved CAR-T cell function through gene delivery into less differentiated T cells followed by their natural physiological expansion, as well as enhanced safety and preservation of the native immunity by avoiding lymphodepletion [4,5].

In vivo CAR-T cell engineering has initially been explored in hematological malignancies and is now expanding toward solid tumors, where conventional CAR-T cell therapies have shown limited efficacy. In addition, engineering approaches are also being extended beyond T cells to other immune cell populations, including natural killer (NK) cells and macrophages. Among solid tumors, malignant glioma represents a critical frontier and is highly resistant to CAR-T cell therapies due to its antigen heterogeneity, immunosuppressive microenvironment, and limited T cell infiltration. Whether in vivo-engineered CAR-based immune cells can overcome these barriers and serve as a transformative approach for the treatment of malignant glioma remains an open and important question.

Although several review articles have previously addressed this topic, the field is evolving rapidly [6,7]. This review provides an updated synthesis of recent technological advances and emerging clinical data, while also offering one of the first focused discussions on the potential application of in vivo CAR-T cell engineering to malignant glioma.

## 2. Methods

Relevant literature was identified through searches of PubMed. PubMed searches were conducted using combinations of the terms “in vivo CAR”, “chimeric antigen receptor”, “CAR-T”, “CAR-NK”, “CAR-macrophage”, “glioma”, “glioblastoma”, “viral vector”, and “nanoparticle”. Representative Boolean queries included (“in vivo” AND (“CAR” OR “chimeric antigen receptor”)) and ((“glioma” OR “glioblastoma”) AND (“CAR-T” OR “CAR-NK” OR “CAR-macrophage”)). Priority was given to peer-reviewed publications published up to May 2026. Additional references were identified through citation screening of relevant articles.

A search of ClinicalTrials.gov was performed on 31 May 2026 to identify clinical trials of in vivo CAR-based immune cell engineering. The database was queried using the Boolean search string (“CAR” OR “chimeric antigen receptor”) AND “in vivo”. Studies with a recruitment status of “Recruiting,” “Active, not recruiting,” or “Completed” were included for further review. For trials in which relevant information was not available through ClinicalTrials.gov, additional study details, including platform characteristics, vector systems, and target antigens, were obtained from publicly available disclosures provided by the corresponding developers. To collect available clinical outcome data, peer-reviewed publications and conference abstracts associated with relevant studies were reviewed when available. Company press releases were excluded from efficacy and safety assessments to minimize potential reporting bias. When multiple sources were available, peer-reviewed publications were prioritized, followed by conference abstracts. Trial status and study characteristics were verified using the most recent publicly available information available at the time of data collection.

## 3. Current Clinical Experience of Ex Vivo CAR-T Cell Therapy in Malignant Glioma

Despite numerous clinical trials evaluating ex vivo engineered CAR-T cell therapies in patients with malignant glioma, durable clinical responses and survival benefits comparable to those achieved in hematological malignancies have remained elusive. A distinctive aspect of CAR-T cell therapy for malignant glioma is the frequent use of locoregional delivery approaches, including intracerebroventricular (ICV) and intratumoral administration, to enhance trafficking to tumors protected by the blood–brain barrier (BBB). For example, in a phase 1 trial evaluating ICV delivery of bicistronic CAR-T cells targeting interleukin-13 receptor alpha 2 (IL13Rα2) and epidermal growth factor receptor (EGFR) in patients with recurrent GBM, 18 patients received treatment. While grade 3 immune effector cell-associated neurotoxicity syndrome (ICANS) occurred in 56% of patients, the objective response rate was only 8% [8]. Similarly, in a phase 1 trial evaluating locoregional delivery of IL13Rα2-targeting CAR-T cells in patients with recurrent high-grade glioma, 57 patients received treatment. Although grade 3 or higher adverse events were observed in 35% of patients, the objective response rate was only 5.2% [9]. In a phase 1 trial of GD2-targeting CAR-T cell therapy for patients with H3K27M-mutant diffuse intrinsic pontine glioma (DIPG), utilizing both intravenous (IV) and ICV administration, 13 patients were enrolled. Although marked tumor reductions of up to 100% were observed in 4 of 13 patients, tumor inflammation-associated neurotoxicity (TIAN) occurred in all patients, and grade 3 or higher cytokine release syndrome (CRS) developed in 50% of patients receiving dose level 2 IV infusion [10]. Overall, limited efficacy and substantial toxicity remain major challenges in CAR-T cell therapy for malignant glioma. In the following section, we discuss the mechanisms underlying resistance to CAR-T cell therapy in malignant glioma.

## 4. Resistance Mechanisms to CAR-T Cell Therapy in Malignant Glioma

The major barriers to effective CAR-T cell therapy in malignant glioma are multifactorial and highly interconnected. Resistance arises from a combination of patient-related factors, the profound local and systemic immunosuppression, antigen heterogeneity and escape, and restricted trafficking across the BBB. These barriers collectively limit CAR-T cell expansion, persistence, and antitumor activity. Therefore, successful in vivo-engineered CAR-T cell therapies for malignant glioma will likely require integrated multimodal strategies rather than approaches targeting individual barriers in isolation. In the following sections, we discuss the major mechanisms underlying resistance to CAR-T cell therapy in malignant glioma.

In GBM, patients receive chemoradiation therapy using temozolomide (TMZ) as the standard of care, which often causes prolonged and profound lymphopenia. Furthermore, lymphocytes in patients with GBM exhibit impaired function, including reduced proliferative capacity, diminished cytokine production, and features of T cell exhaustion. Moreover, patients with malignant glioma are often treated with dexamethasone to manage cerebral edema, which has been shown to induce cytotoxic T-lymphocyte-associated protein 4 (CTLA-4) expression, particularly in naïve T cells, thereby inhibiting their proliferation and differentiation and contributing to T cell dysfunction [11]. Even in treatment-naïve settings, the presence of brain tumors has been shown to induce the loss of S1P1 receptors from the T cell surface, leading to the sequestration of large numbers of T cells in the bone marrow [12]. In addition, GBM predominantly affects elderly patients, and age-associated T cell dysfunction may represent an additional barrier [13,14]. All of these mechanisms result in a reduced pool of functional T cells in patients with malignant glioma.

The immunosuppressive tumor microenvironment and antigen heterogeneity represent two major mechanisms of resistance to CAR-T cell therapy in malignant glioma. In GBM, abundant infiltration of immunosuppressive myeloid cells, including myeloid-derived suppressor cells (MDSCs), together with hypoxic and nutrient-deprived conditions, creates a profoundly immunosuppressive tumor microenvironment that impairs and exhausts tumor-infiltrating CAR-T cells [15,16,17,18,19]. In addition, no currently identified target antigen is both tumor-specific and homogeneously expressed across tumor cells. Consequently, antigen heterogeneity remains a major obstacle to the effective implementation of CAR-T cell therapy. Furthermore, epidermal growth factor receptor variant III (EGFRvIII), one of the most extensively studied tumor-specific antigens in GBM, has been reported to be lost at recurrence following CAR-T cell therapy, highlighting the challenge of antigen escape [20].

The trafficking of T cells to the brain is another major challenge. The brain is protected by the BBB. Although activated and central nervous system (CNS) antigen-specific T cells can cross the BBB and migrate into the brain parenchyma, T cells, in general, do not readily do this. In recent clinical trials of ex vivo CAR-T cell therapy for GBM, ICV delivery has been selected over IV administration to improve CAR-T cell delivery to the tumor [9,21,22,23], although the CSF space is separated from the brain parenchyma by the glia limitans.

Collectively, these challenges suggest that the limitations in developing in vivo-engineered CAR-T cell therapies for malignant glioma are not attributable to a single barrier but rather to the convergence of multiple factors, including intrinsic T cell properties and restricted trafficking across the BBB. Addressing these limitations will likely require integrated strategies that combine optimized vector design, enhancement of trafficking capacity, alternative immune cell platforms such as CAR-macrophages, and approaches to transiently modulate the BBB. A comprehensive understanding of these barriers and their interplay will be essential for the rational development of in vivo-engineered CAR-based immune cell therapies for malignant glioma.

## 5. Limitations of Current Ex Vivo CAR-T Cell Manufacturing

**Figure 1:**
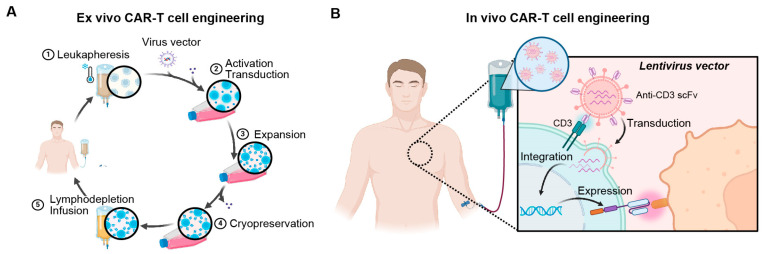
Overview of ex vivo and in vivo CAR-T cell engineering approaches. (**A**) Conventional ex vivo CAR-T cell engineering. Autologous T cells are collected by leukapheresis, genetically modified ex vivo using viral vectors to introduce a CAR transgene, expanded, cryopreserved, and subsequently infused into the patient following lymphodepleting chemotherapy. (**B**) In vivo CAR-T cell engineering. Gene delivery vectors are administered directly to the patient and selectively transduce endogenous T cells in vivo. Following transduction, CAR transgenes are expressed within host T cells, enabling the generation of CAR-T cells without ex vivo cell manufacturing. The representative example shown depicts a lentiviral vector displaying an anti-CD3 targeting moiety that mediates T cell transduction and stable CAR expression through genomic integration. All schematics were created using BioRender.com.

All seven Food and Drug Administration (FDA)-approved CAR-T cell products require autologous ex vivo CAR-T cell engineering, and previous clinical trials of CAR-T cell therapy for malignant glioma have similarly employed this approach (Figure 1A). Ex vivo autologous CAR-T cell therapy poses a myriad of challenges; its multi-step manufacturing workflow, driven by individualized production, is labor-intensive, time-consuming, requires specialized facilities, and significantly increases costs, making therapy inaccessible to many patients. Approved CAR-T products are estimated to cost over USD 500,000 per patient, and the total cost of CAR-T cell therapy, including associated treatment expenses, is reported to exceed USD 1–1.5 million [24]. Manufacturing also takes weeks and is inconvenient for patients with rapidly growing tumors who face time constraints.

Furthermore, because current FDA-approved methods utilize lentiviral (LV) or retroviral vectors to permanently integrate the CAR transgene into patient T cells, the CAR is continually expressed, making the cells more prone to exhaustion and differentiation due to tonic signaling and cytokine exposure [25,26]. Long periods of in vitro manipulation and expansion, and the nonimmediate infusion of product cells into the patient, can increase T cell exhaustion and differentiation, which reduces persistence and long-term efficacy. Expanding cells with interleukins (IL)-7 and IL-15, and selecting less differentiated central memory and stem cell memory T cells for patient infusion, are strategies used to mitigate exhaustion and differentiation [9,27,28].

Additionally, manufacturing variability arises because the starting material depends on the patient’s immune status; heavily pretreated or immunocompromised patients often yield low-quality T cells, which can pose challenges for transduction, expansion, and functional assessment during ex vivo genetic modification, leading to inconsistent therapeutic outcomes. A method is needed to ensure that every patient receives efficacious treatment.

Lymphodepletion prior to infusion is also needed to prevent host immune rejection and create a favorable environment for CAR-T cell expansion and persistence [29,30]. However, lymphodepletion poses a high risk of immune system suppression, severe infections, cytopenias (such as neutropenia and anemia), hematological toxicities (including prolonged bone marrow suppression, which delays hematopoietic recovery), CRS, and neurotoxicity [31]. Patients who have higher tumor burden, older age, or have undergone previous chemotherapy lines are even more vulnerable to complications. These risks must be closely monitored during the peri-infusion period, particularly for CRS and ICANS, the two most common and potentially fatal complications.

The limitations described above are well recognized as challenges in conventional ex vivo CAR-T engineering, underscoring the growing need for in vivo CAR-T cell engineering approaches. In the following section, we discuss the potential advantages of in vivo CAR-T cell engineering in addressing these issues.

## 6. Potential Advantages of In Vivo CAR-T Cell Engineering

To overcome the intrinsic limitations of ex vivo engineered CAR-T cell therapy, an in vivo-engineered CAR-T approach has been developed as a promising approach to directly reprogram host T cells by introducing CAR transgenes (Figure 1B). This approach relies on the mass production of targeted gene-delivery vehicles, such as viral vectors and nanoparticles, to encapsulate CAR transgenes. CAR transgene-containing vectors are synthesized and rigorously validated at scale in centralized facilities, thereby bypassing the lengthy manufacturing and quality control steps in ex vivo CAR-T manufacturing. These products can be stocked as off-the-shelf therapeutics and may enable more rapid initiation in response to clinical demand. In some settings, this approach could substantially shorten the therapeutic timeline compared with conventional ex vivo CAR-T cell manufacturing, particularly for rapidly progressive tumors. The scalable, centralized manufacturing approach may reduce manufacturing costs and improve accessibility compared with conventional ex vivo CAR-T cell therapies. Manufacturing costs have been projected to decrease to approximately USD 5000 per dose; however, this estimate remains preliminary and will require validation in mature clinical and commercial settings [32].

The need for off-the-shelf products has increased in recent years, and allogeneic CAR-T approaches are being actively developed, including the donor-derived CAR-T cells in which endogenous T cell receptor (TCR) and major histocompatibility complex (MHC) genes are disrupted using CRISPR/Cas9, as well as CAR-T cells derived from induced pluripotent stem cells (iPSCs) [33,34,35,36]. Although allogeneic CAR-T cell therapy may represent a useful approach for patients with malignant glioma, in whom the T cell pool is reduced and T cell function is impaired, allogeneic CAR-T cell therapies are associated with several challenges, including potential impairment of cellular fitness due to extensive genome editing, reduced persistence resulting from host immune clearance, and safety risks such as graft-versus-host disease (GVHD) [37]. In contrast, in vivo-engineered CAR-T cell therapies, which are autologous, avoid these limitations.

In vivo CAR-T cell engineering can offer an improved safety and efficacy profile, since it directly delivers CAR transgenes into endogenous immune cells and therefore does not require complete lymphodepletion. Non-viral platforms, such as lipid nanoparticles (LNPs), further reduce these risks, since transient CAR expression and mRNA stability act as a safety switch to limit acute cytokine storm and mitigate chronic toxicities [38]. Beyond safety profiles, avoiding lymphodepletion can better preserve patients’ overall immune system integrity. An intact immune system will enhance tumor epitope spreading, enabling recognition of additional tumor-associated antigens beyond the initial CAR target [39]. This process supports broader antitumor activity and mitigates tumor heterogeneity and antigen escape, two major challenges in solid tumors [39,40].

Another fundamental biological advantage of in vivo CAR-T cell engineering lies in its ability to reprogram T cells in their native physiological niche, thereby preserving a broader, less differentiated T cell repertoire. By skipping ex vivo expansion, unstimulated cells retain superior intrinsic fitness and enhanced antitumor activity [4,41]. This advantage is also associated with enhanced cell expansion and sustained antitumor effects at lower doses than conventional CAR T therapies [42].

Despite these advantages, achieving precise cell-type specificity remains a central challenge for in vivo CAR-T cell engineering. Both viral and non-viral delivery systems need to be carefully designed to selectively target immune cells while minimizing off-target transduction in the liver and spleen, where viral and nanoparticle vectors accumulate [43,44]. Strategies to enhance targeted delivery include engineering vector surfaces to express T cell-specific binding molecules, such as anti-CD3 scFv, anti-CD7 scFv, and anti-TCR nanobodies, as well as developing adeno-associated virus (AAV) serotypes capable of selectively transducing T cells through capsid screening [45,46]. Furthermore, off-target toxicity can be mitigated by bioengineering viral and nanoparticle vectors to evade innate immune clearance, such as by incorporating CD47 into the vector surface, which reduces nonspecific macrophage-mediated phagocytosis [47]. Toxicity resulting from off-target transduction by vectors with broad tropism may represent a major safety concern. In the context of malignant glioma, off-target transduction of glial cells, including microglia, as well as neurons, can induce unexpected neuroinflammation, peritumoral edema, and seizures, all of which may be further exacerbated in this setting.

The limitations of conventional ex vivo CAR-T engineering and the advantages offered by in vivo CAR-T cell engineering approaches have been discussed (Table 1). The following sections will focus on the latest technologies, transgene delivery modalities, and expression types currently employed in in vivo CAR-T cell engineering. In addition, Section 7 reviews the currently available clinical data on in vivo-engineered CAR-T cell therapy, highlighting both the therapeutic promise and the safety challenges observed in early clinical studies.

## 7. Transient Expression vs. Stable Expression, Site-Specific Integration

CAR expression in T cells falls into two categories: transient and stable, each defined by expression dynamics, distinct delivery mechanisms, and clinical use cases. The choice between these approaches is guided by the intended duration of CAR activity, safety considerations, and whether the application is early-stage exploration or full therapeutic deployment.

Highly efficient transient CAR expression in T cells via non-viral approaches, including LNPs, polymeric nanoparticles, and membrane-derived particles, is most often achieved by CAR-encoding mRNA [48]. This non-integrating method enables rapid CAR expression, detectable within hours, peaking at approximately 24 to 48 h, and declining over several days as the mRNA is degraded and diluted through cell division. Such short-lived expression provides important safety advantages, particularly in early-phase clinical studies or when targeting antigens with uncertain on-target/off-tumor effects. For example, targets such as GD2 have been associated with neuroinflammatory toxicity, making transient expression a useful strategy to mitigate potential risks [49,50]. In addition, transient systems are well-suited for rapid construct screening and iterative design cycles, enable cost-effective, fast manufacturing, and are subject to fewer regulatory constraints.

However, this safety advantage comes at the expense of durability. Limited persistence of mRNA-based CAR expression may impair long-term CAR-T cell persistence, memory formation, and durable antitumor immunity, potentially reducing the ability to control infiltrative or recurrent disease. In addition, insufficient persistence may limit epitope spreading and endogenous immune activation within the glioma microenvironment. Because repeated dosing may be required to maintain therapeutic efficacy, concerns regarding anti-vector humoral immune responses, reduced delivery efficiency, cumulative neuroinflammation, and treatment-related toxicity remain important considerations, particularly for CNS-directed applications. Furthermore, repeated dosing may be insufficient to support the formation of durable tissue-resident memory T cells, potentially limiting long-term immune surveillance and disease control.

In contrast, stable CAR expression relies on long-term maintenance of the CAR transgene through random genomic integration, supporting prolonged T cell persistence and antitumor activity. Lentiviral and γ-retroviral vectors remain the most widely used delivery platforms and serve as the basis for all FDA-approved CAR-T cell therapies to date. In contrast, in vivo CAR-T cell engineering is achieved using surface-engineered lentiviral vectors with enhanced cell-type specificity, enabling selective delivery of the CAR transgene into the target immune cell population.

Despite their therapeutic advantages, stable expression platforms introduce additional challenges, including more complex and time-intensive manufacturing, higher production costs, and risks inherent to genomic integration, such as insertional mutagenesis and dysregulated gene expression. Recent regulatory safety communications have highlighted the potential risk of secondary T-cell malignancies, including T-cell lymphoma, following ex vivo-manufactured CAR-T cell therapy [51]. Although several cases of CAR-positive T-cell lymphoma have been reported, recent registry analyses suggest that the overall incidence remains extremely low. For example, recent reviews by the European Medicines Agency (EMA) identified 38 reported cases of T-cell malignancies among approximately 42,500 patients treated with CAR-T cell products, with only a subset considered potentially related to the CAR transgene itself [51,52]. Based on the available evidence, current regulatory recommendations emphasize long-term patient monitoring, integration-site analysis when feasible, and continued post-marketing surveillance rather than restriction of lentiviral CAR-T cell therapies [51,52]. Nevertheless, these findings underscore the importance of carefully evaluating insertional mutagenesis risks in in vivo CAR-T cell engineering approaches. In contrast to ex vivo engineered CAR-T cell products, in vivo platforms may result in a greater number of integration events and may also increase the risk of off-target transduction in non-T-cell populations. Such risks may be particularly relevant for integrating viral vectors with broad tropism, highlighting the need for improved cell-type specificity, controlled transgene expression, and long-term safety assessment during future clinical development.

Furthermore, prolonged CAR-T cell persistence may increase the likelihood of chronic toxicities, including CRS and sustained on-target/off-tumor effects. To address these concerns, advanced design strategies, such as inducible safety switches and tunable regulatory elements, are increasingly incorporated to modulate CAR activity and improve safety profiles. Collectively, transient and stable CAR expression systems represent complementary approaches, with ongoing advances in genome engineering continuing to refine their balance between safety, efficacy, and durability.

More recently, genome editing technologies, particularly CRISPR/Cas9, have enabled targeted integration of CAR transgenes into defined genomic loci, most notably the T cell receptor alpha constant (TRAC) locus, resulting in more uniform expression while simultaneously disrupting endogenous TCR signaling. This strategy has been associated with reduced tonic signaling, decreased exhaustion, and improved functional persistence [53]. Furthermore, it may reduce the risk of insertional mutagenesis associated with random genomic integration. Beyond the TRAC locus, alternative genomic loci have been explored to further refine CAR-T cell function. Other genomic “safe harbor” loci, such as adeno-associated virus integration site 1 (AAVS1) and ROSA26, have also been explored. These loci provide alternative platforms for standardized CAR insertion, particularly in contexts where preservation of endogenous TCR function may be desirable [54]. Furthermore, emerging tools, including non-viral delivery systems and alternative nucleases like Cas12a, continue to expand the precision engineering landscape [55]. Recent studies have demonstrated that these genome-editing approaches can be combined with T-cell-tropic delivery platforms, including engineered AAV serotypes and Cas9-containing enveloped delivery vehicles (EDVs), to enable locus-specific CAR integration directly within endogenous T-cell populations in vivo [56]. While targeted integration may reduce the risk of insertional mutagenesis, it also introduces distinct safety considerations, including off-target genome editing, unintended disruption of endogenous gene function, and double-strand break-associated genomic instability, such as large deletions, chromosomal rearrangements, and translocations [57,58]. Careful evaluation of these risks will be essential as in vivo genome-editing approaches advance toward clinical application.

## 8. In Vivo CAR-T Cell Engineering Platforms

**Figure 2:**
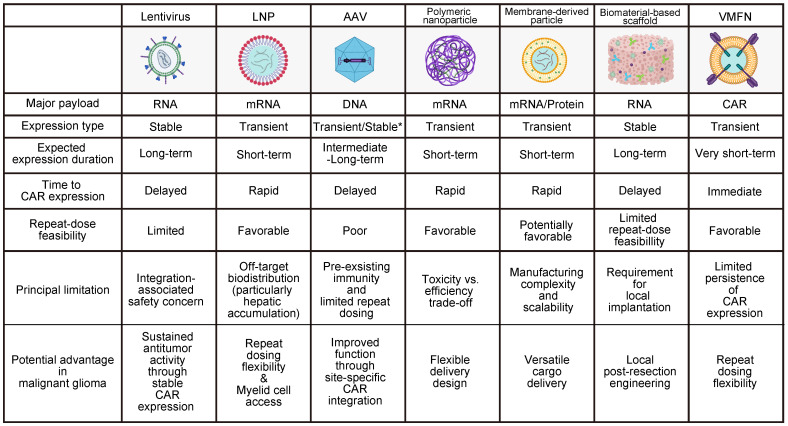
This schematic summarizes the key characteristics and principal limitations of representative vector platforms used for in vivo engineering, including lentivirus, lipid nanoparticle (LNP), adeno-associated virus (AAV), polymeric nanoparticle, membrane-derived particle, biomaterial-based scaffold, and virus-mimetic fusion nanoparticles (VMFNs). * Although AAV-mediated expression is generally transient owing to its episomal DNA, stable expression has been achieved in combination with Cas9-enveloped delivery vehicle (EDV). The schematic was created using BioRender.com.

Several vector platforms applicable to in vivo CAR-T cell engineering have been developed. Among these, LV vectors and LNPs currently represent the most clinically advanced, as reflected by their use in ongoing clinical trials of in vivo-engineered CAR-based immune cell therapies. Nevertheless, each platform offers distinct advantages and limitations in terms of targeting specificity, expression kinetics, persistence, safety, and repeat-dose feasibility. It should be noted, however, that malignant glioma-specific preclinical data remain limited or unavailable for most of these platforms, and their applicability to malignant glioma has yet to be fully established. In this section, we discuss the characteristics, translational potential, and remaining challenges of each platform in detail (Figure 2).

### 8.1. Lentivirus

LV vectors are single-stranded RNA viruses derived from retroviruses that are widely used for gene transfer in CAR-T cell engineering [59]. Owing to their ability to transduce both dividing and non-dividing cells and mediate stable genomic integration, LV vectors enable long-term CAR expression and sustained antitumor activity. As a result, they currently represent the most clinically advanced platform for both ex vivo and emerging in vivo CAR-T cell engineering approaches [60,61]. In addition, their established Good Manufacturing Practice (GMP) production pipelines and broad clinical experience have contributed to their high clinical readiness and translational applicability.

Recent advances have significantly improved the targeting specificity of LV-based in vivo CAR delivery systems. By incorporating alternative viral envelope proteins derived from Sindbis, Nipah, or measles viruses, researchers have engineered vectors that selectively target defined immune cell subsets, including CD3^+^, CD4^+^, CD7^+^, and CD8^+^ T cells [7,62]. These systems frequently employ targeting ligands, such as scFvs or designed ankyrin repeat proteins (DARPins) fused to viral glycoproteins, thereby reducing off-target transduction and improving therapeutic specificity and antitumor efficacy [62]. Furthermore, LV vectors engineered to express multidomain fusion (MDF) proteins, including anti-CD3 scFv fused with costimulatory ligands such as CD58 and CD80, have been reported to further enhance in vivo CAR transduction efficiency and antitumor activity [63].

Despite these advantages, important safety concerns remain. Although the risk of insertional mutagenesis associated with LV vectors has been discussed in the previous section, concerns regarding replication-competent virus generation and prolonged off-target transduction remain important considerations, particularly for CNS applications [64]. In addition, larger transgene constructs may reduce functional vector titers, highlighting a trade-off between targeting specificity, payload complexity, and manufacturing efficiency [62,65]. Collectively, LV vectors currently represent the most clinically mature in vivo CAR delivery platform, although continued optimization of safety, targeting precision, scalability, and CNS applicability remains necessary.

### 8.2. Lipid Nanoparticle

LNP-mediated delivery has rapidly emerged as a leading non-viral strategy for in vivo CAR-based immune cell engineering, largely driven by advances in mRNA therapeutics. Recent studies have demonstrated that LNPs enable direct systemic delivery of CAR-encoding mRNA or DNA, thereby allowing transient in vivo T cell reprogramming [7]. Unlike viral vector integration, LNP systems mediate transient CAR expression, which may allow greater control over CAR activity, toxicity, and repeat dosing [66,67].

Recent advances in antibody-conjugated targeted LNPs have significantly improved targeting specificity, including selective delivery to CD8^+^ T cells [67]. In addition, LNP systems offer major advantages in scalability and manufacturability because they are synthetically produced, modular, and rapidly redesignable, potentially reducing vein-to-vein time and manufacturing costs compared with conventional ex vivo CAR-T cell production [68]. These advantages have driven substantial industry interest, with LNP-based systems representing a rapidly expanding proportion of newly disclosed in vivo CAR-based immune cell engineering platforms [69].

However, several important limitations remain. Efficient and highly selective delivery to T cells in vivo remains challenging, particularly because LNPs frequently accumulate in the liver and other off-target tissues [67]. Interestingly, this biodistribution profile may also be advantageous, as preferential uptake by myeloid populations has been exploited for in vivo engineering of macrophages and other myeloid cells, supporting the development of CAR-myeloid cell-based therapies for solid tumors. Furthermore, transient CAR expression may limit long-term persistence and antitumor efficacy in oncology applications, potentially necessitating repeated administration. Repeated dosing may also increase risks of immunogenicity and anti-drug antibody formation. Nevertheless, the controllable, non-integrating nature of LNP systems may provide important safety advantages for CNS malignancies, where reversible CAR expression could reduce the risk of prolonged neurotoxicity.

### 8.3. AAV

AAV vectors are non-pathogenic, single-stranded, non-enveloped DNA viruses that have been widely utilized as gene delivery platforms in both preclinical and clinical settings [70]. Following cellular entry, the delivered genome is predominantly maintained as episomal DNA within the nucleus, enabling relatively durable transgene expression while minimizing the risk of insertional mutagenesis compared with integrating viral vectors [70]. Owing to their favorable safety profile and long-term expression characteristics, AAV vectors have emerged as promising tools for in vivo gene transfer applications [71].

A major advantage of AAV vectors is their diverse capsid serotypes, which confer distinct tissue tropisms and enable selective targeting of specific cell populations [46,56,65,66,72,73]. These characteristics make AAV vectors attractive for applications requiring durable transgene expression and precise cellular targeting.

However, considerable limitations remain. The relatively small packaging capacity of AAV (~4.7 kb) restricts the size and complexity of CAR constructs and associated regulatory elements [74]. In addition, pre-existing immunity against AAV capsids may substantially reduce transduction efficiency and limit the feasibility of repeated administration [75]. Prolonged persistence of episomal genomes may also complicate precise control of CAR expression and raise concerns regarding prolonged off-target transduction and immune-related toxicities, particularly in CNS tissues [76]. Thus, although AAV vectors exhibit favorable safety profiles and established clinical readiness, further optimization of payload flexibility, repeat-dose feasibility, and CNS-specific targeting remains necessary.

### 8.4. Polymeric Nanoparticle

In addition to LNPs, polymer-based nanoparticles are also widely utilized for in vivo CAR-T cell engineering. Compared with LNP systems, polymeric nanoparticles exhibit greater structural diversity and offer a more flexible delivery design, with controllable size, charge, and surface functionality, thereby enabling optimization of circulation time and cell-specific targeting [77]. Cationic polymers, such as polyethylenimine (PEI) and poly(β-amino ester) (PBAE), can electrostatically bind negatively charged nucleic acids and facilitate gene loading and intracellular release. Surface modifications, including poly(ethylene glycol) (PEG) coating, can further improve biocompatibility and prolong systemic circulation by reducing opsonization and immune clearance [78].

Targeting specificity can be enhanced through the conjugation of antibody fragments or targeting ligands. For example, anti-CD3ε F(ab’)2 fragments have enabled selective transduction of T cell populations [79]. Additional engineering strategies incorporating microtubule-associated sequences and nuclear localization signals have also improved intracellular trafficking and nuclear delivery efficiency [80]. These modular properties make polymeric nanoparticles attractive candidates for scalable and customizable in vivo CAR delivery platforms.

Nevertheless, several limitations remain. Compared with viral vectors, polymeric nanoparticles generally exhibit lower transfection efficiency [81]. In addition, off-target uptake by macrophages, monocytes, and B cells within the liver and spleen remains a significant challenge [81]. Although PEGylation improves biocompatibility, repeated administration may induce anti-PEG antibodies, thereby reducing therapeutic efficacy [82]. Therefore, while polymeric nanoparticle systems offer substantial advantages in modularity, manufacturability, and scalability, further improvements in targeting specificity and delivery efficiency will be necessary to fully realize their clinical potential for CNS malignancies.

### 8.5. Membrane-Derived Particle

Membrane-derived particles, including extracellular vesicles (EVs) [83] and virus-like particles (VLPs) [73], represent biologically derived nanocarriers that utilize the structural and functional properties of cellular membranes. These particles are composed of lipid bilayers enriched with membrane proteins and can encapsulate diverse molecular cargoes, including nucleic acids and proteins.

One major advantage of membrane-derived particles is their high biocompatibility and relatively low immunogenicity compared with synthetic nanoparticles and viral vectors. Their surfaces can also be engineered to display targeting ligands such as antibodies, scFvs, or nanobodies, thereby improving targeting specificity and programmable delivery. EVs engineered with anti-CD3 nanobodies have demonstrated antitumor activity comparable to LNPs and LV vectors while maintaining favorable immunogenicity profiles [83]. In addition, these systems are particularly attractive for protein delivery, including Cas9 ribonucleoprotein complexes, owing to their ability to preserve protein structure and facilitate intracellular trafficking.

Cas9-EDVs further expand the applicability of this platform by enabling T cell-selective, site-specific CAR integration via scFv display targeting CD3, CD4, or CD28 [73,84]. Furthermore, membrane fusion properties may partially bypass endosomal degradation pathways, thereby improving cytosolic delivery efficiency.

However, several significant challenges remain. Heterogeneity in particle composition may result in inconsistent cargo loading and variable delivery efficiency. In addition, scalable GMP-compatible manufacturing and reproducible production remain major barriers to clinical translation. Precise control of biodistribution and targeting specificity also remains limited, and unintended off-target delivery cannot be fully excluded. Consequently, although membrane-derived particles represent a promising biologically inspired delivery platform with potentially favorable safety characteristics, further optimization of production scalability, cargo loading efficiency, and targeting precision will be required.

### 8.6. Biomaterial-Based Scaffolds

Biomaterial-based scaffolds were originally developed as localized depots for the sustained release of therapeutic agents, including small molecules, proteins, and vaccines, in order to improve pharmacokinetics while minimizing systemic toxicity. More recently, advances in immune engineering have repurposed these scaffolds as platforms for localized immune modulation by creating transient microenvironments enriched with cytokines, antigens, and stimulatory signals [85,86].

Injectable hydrogels and porous scaffolds composed of materials such as collagen and alginate have subsequently been applied to both ex vivo and in vivo CAR-T cell engineering. These systems can recruit immune cells, support lentiviral-mediated engineering of endogenous T cells, promote local T cell expansion, and enable controlled release toward tumor sites. For example, porous collagen scaffolds functionalized with the C-C motif chemokine ligand (CCL) 21 and incorporating lentiviral vectors encoding CAR transgenes, together with stimulatory anti-CD3/CD28 antibodies, have been developed to recruit endogenous T cells and enable their localized genetic reprogramming into CAR-T cells [85].

A major advantage of biomaterial-based scaffolds is their ability to deliver drugs locally and spatially, while potentially minimizing systemic toxicity. These properties may be particularly attractive for CNS malignancies, where locoregional delivery approaches are increasingly important. However, clinical readiness remains relatively limited compared with viral or LNP-based systems, and challenges regarding scalability, reproducibility, long-term biocompatibility, and manufacturing standardization remain unresolved. Further optimization of biomaterial composition, immune modulation, and delivery kinetics will therefore be required for broader translational application.

### 8.7. Virus Mimetic-Fusogenic Nanovesicle (VMFN)

VMFNs are engineered lipid membrane nanovesicles expressing modified viral fusogens derived from reovirus or measles virus fused with anti-CD3 scFvs [87]. In addition, CAR proteins displayed on the vesicle surface are directly transferred to target cell membranes following membrane fusion. Unlike LV systems that primarily utilize endocytic pathways, these fusogenic systems mediate direct plasma membrane fusion, thereby enabling transient protein-level CAR transfer.

A major advantage of this platform is its transient expression profile, which avoids genomic integration and thereby eliminates the risk of insertional mutagenesis. CAR expression is rapidly induced following fusion, peaks approximately 24 h later, and gradually declines thereafter. In preclinical studies, CAR-positive T cells accounted for approximately 2–3% of circulating T cells following administration, and antitumor efficacy was demonstrated in murine lymphoma and rheumatoid arthritis models [88]. The transient and non-integrating nature of this system may provide important safety advantages, particularly for CNS malignancies, where prolonged CAR activity may increase the risk of chronic neurotoxicity or neuroinflammation.

In addition, targeting specificity can be improved by incorporating anti-CD3 scFvs and fusogenic proteins, while the absence of viral replication machinery may reduce immunogenicity and regulatory complexity compared with integrating viral vectors. However, clinical readiness remains early, and important limitations include relatively low transduction efficiency, transient persistence, limited manufacturing experience, and uncertain scalability. Further optimization of delivery efficiency, persistence, and reproducible manufacturing will therefore be necessary before clinical translation can be fully realized.

## 9. Clinical Studies Evaluating In Vivo-Engineered CAR-Based Immune Cells

Several clinical trials of in vivo-engineered CAR-based immune cell therapies have already been initiated in hematologic malignancies, and preliminary clinical data from some of these studies have begun to emerge. At present, LV vectors represent the predominant gene-delivery platform used in clinical studies of in vivo CAR engineering (Table 2), although early clinical investigations utilizing LNP have also been initiated (Table 3). As the scope of in vivo CAR engineering expands beyond T cells to include other immune cell types, clinical trials targeting solid tumors have also been initiated. Furthermore, in vivo-engineered CAR-based immune cell therapies are being investigated not only for malignant diseases but also for autoimmune disorders. However, this field is rapidly evolving, and several platforms remain proprietary, limiting the availability of detailed technical information for some ongoing clinical programs.

Within this context, interim analysis data from a phase 1 trial of ESO-T01, a LV vector product encoding a B cell maturation antigen (BCMA)-targeting CAR transgene, have been reported in patients with relapsed or refractory multiple myeloma [90]. ESO-T01 is designed to enable T cell-specific delivery of the CAR transgene via an anti-TCR nanobody. A total of five patients were enrolled, with a median follow-up duration of 6 months. The median time from enrollment to infusion was 8 h and 37 min. The proportion of CAR-T cells among CD3^+^ T cells reached 10.6–59.1% by day 14 post-infusion. Regarding clinical responses, the overall response rate was 80%, including three stringent complete responses and one partial response, all of which were maintained throughout the follow-up. Grade 3 CRS occurred in 60% of patients. Cytopenia was the most common adverse event, with grade 3 lymphopenia observed in all patients and grade 3 elevations in aspartate aminotransferase (AST) in 80% patients. Although based on a limited number of reports, this approach suggests logistical feasibility and has demonstrated preliminary but robust CAR-T cell expansion and encouraging short-term efficacy. However, further evaluation is required to assess long-term safety, efficacy, and CAR-T cell persistence, including the potential risk of oncogenic mutations associated with random LV vector integration. Significant uncertainties regarding safety remain. Within 24 h after LV vector administration, elevations in IL-6, IL-8, tumor necrosis factor (TNF), and ferritin, as well as cytopenias, were observed, suggesting a hyperacute inflammatory reaction potentially driven by innate immune recognition of the LV vector. In addition, a fatal case of spinal cord compression associated with enlargement of extramedullary disease during the CAR-T cell expansion phase was reported. It remains unclear whether this event reflected true disease progression, pseudoprogression, or inflammatory edema. Further studies are therefore needed to clarify the mechanisms underlying these adverse events and their impact on both safety and therapeutic efficacy. Moreover, important clinical considerations, including pre-treatment strategies, patient selection, and vector design, will require careful optimization as these platforms advance toward broader clinical application.

A phase 1 study evaluating the safety and preliminary efficacy of a single dose of KLN-1010, another in vivo CAR-T cell engineering platform, is currently underway in patients with refractory multiple myeloma (NCT07075185). KLN-1010 is a third-generation LV vector engineered to selectively deliver the CAR transgene to T cells through surface expression of a modified vesicular stomatitis virus glycoprotein (VSV-G) fusogen and an anti-CD3 antibody. An interim analysis of the first three treated patients has been reported [89]. Robust in vivo expansion was observed, with CAR expression detected in 22–72% of CD3^+^ T cells at day 15 post-infusion despite the absence of lymphodepleting preconditioning. As this platform utilizes an LV vector, the CAR transgene is integrated into the host genome. Notably, 51,647–65,873 copies of the CAR transgene per μg of genomic DNA were detected in blood and bone marrow samples 3 months after administration. These levels are comparable to those observed in clinical trials of ex vivo-engineered CAR-T cell therapies, suggesting sustained persistence of in vivo-engineered CAR-T cells. Regarding safety, grade 2 CRS occurred in two patients; however, no cases of ICANS were observed, and cytopenias were limited. Regarding clinical responses, all patients achieved a partial response (PR), with the best response being a very good partial response (VGPR) at 3 months, and no disease progression was observed during follow-up. However, these findings are based on very early clinical experience from only three patients enrolled in the ongoing phase I study. Therefore, additional follow-up and larger patient cohorts will be required to establish the safety, durability, and clinical efficacy of this approach.

Similarly, VivoVec is an LV vector platform that expresses cocal glycoprotein and an MDF protein composed of an anti-CD3 scFv, CD58, and CD80, enabling the selective delivery of the CAR transgene to CD3-positive cells and the efficient activation of these cells [45,63,94]. Cocal glycoprotein has a structure similar to that of VSV-G but is less susceptible to inactivation in vivo, thereby improving persistence [95]. Phase I studies are currently underway evaluating UB-VV400, carrying a CD22-targeting CAR payload, and UB-VV110, carrying a CD19-targeting CAR payload, both based on the VivoVec platform, for the treatment of non-Hodgkin lymphoma and B cell lymphoma, respectively (NCT06743503 and NCT06528301).

In addition, INT2104 is an LV vector engineered to express Generation 2.1 (Gen 2.1) fusogen and a CD7 antibody, enabling selective delivery of the CAR transgene to CD7-positive cells [96]. Gen2.1 is an optimized VSV-G variant that incorporates the single amino acid substitution I182E to detarget VSV-G from the low-density lipoprotein receptor (LDL-R), thereby reducing the risk of off-target transduction in unintended cells, along with the T214N and T352A substitutions to enhance resistance to serum inactivation. Targeting CD7 allows the generation of both CAR-T and CAR-NK cells in vivo. In a preclinical study in cynomolgus macaques, CAR protein expression was detected exclusively in T cells and NK cells following IV administration of INT2104. B-cell depletion was observed by day 4 after administration, with no apparent toxicity. A phase I study in patients with B-cell malignancies is currently underway (NCT06539338).

Beyond LV approaches, non-viral platforms are also being explored. MT-302 is an LNP formulation encapsulating mRNA encoding a TROP-2 CAR, in which the LNP is composed of an ionizable cationic lipid, a PEG lipid, helper lipids, and cholesterol [97]. Unlike the previously described CD3- or CD7-targeted LV vectors, MT-302 delivers mRNA into macrophages via passive targeting by exploiting the natural tropism of LNPs for macrophages. In a preclinical study using mouse models, the frequency of Trophoblast cell surface antigen 2 (TROP2) CAR-positive cells 24 h after MT-302 administration was highest in monocytes, followed by dendritic cells, while little to no CAR expression was detected in other immune cell populations, indicating preferential delivery of the CAR to myeloid cells. In addition, in an HCC-1954 breast cancer xenograft mouse model, five doses of MT-302 administered every four days demonstrated antitumor efficacy without observable adverse events. A phase 1 clinical trial evaluating the safety and tolerability of repeated MT-302 dosing in patients with metastatic epithelial tumors is currently ongoing (NCT05969041) [92]. A total of 27 patients were enrolled and treated. Although the median follow-up duration had not been disclosed at the time of the interim analysis, TROP2 CAR expression was detected in circulating myeloid cells within hours after dosing, and CAR-expressing myeloid cells were shown to co-localize with tumor cells in tumor specimens. Notably, CAR-expressing myeloid cells were shown to infiltrate tumors and activate endogenous immune responses, which is of considerable interest. Regarding safety, CRS occurred in 52% of patients; however, no Grade 3 or higher CRS events were observed, while one patient in the highest-dose cohort developed Grade 4 ICANS. Regarding clinical responses, the best response was a PR lasting 16 months. It should be noted that these findings are based on very early clinical experience from the phase 1 trial of MT-302, and further updates from the ongoing study are anticipated. Interestingly, similar findings have also been reported with ex vivo engineered CAR-macrophage therapy. In a first-in-human phase I trial of CT-0508, an anti-HER2 CAR-macrophage product, 14 patients with advanced HER2-positive solid tumors were treated [98]. No Grade 3 or higher CRS or ICANS was observed, and disease stabilization was achieved in 44% of patients with HER2 3^+^ tumors. In addition, CAR-macrophages trafficked to tumor sites and were associated with remodeling of the tumor microenvironment, including expansion of intratumoral CD8^+^ T cells. These observations support the concept that CAR-engineered myeloid cells may not only exert direct antitumor effects but also enhance endogenous antitumor immune responses.

Using a similar approach to MT-302, a Phase 1 study of MT-303, an LNP formulation encapsulating mRNA encoding a Glypican-3 (GPC3) CAR for hepatocellular carcinoma, and MT-304, an LNP formulation encapsulating mRNA encoding Human epidermal growth factor receptor 2 (HER2) CAR, are also underway (NCT06478693 and NCT07334119).

Taken together, in hematologic malignancies, in vivo-engineered CAR-T cell approaches have begun to demonstrate feasibility and therapeutic promise; however, current clinical data remain early and limited, and several challenges related to delivery efficiency, target specificity, and safety persist. Although clinical trials in solid tumors have also been initiated, additional challenges are anticipated in brain tumors, which will be discussed in the following section.

## 10. Challenges of Applying In Vivo-Engineered CAR-Based Immune Cell Therapies to Malignant Glioma

As discussed in Section 3, multiple resistance mechanisms limit the efficacy of CAR-based immune cell therapies in malignant glioma. While in vivo CAR-based immune cell engineering may offer several unique advantages for this disease, it also introduces distinct challenges that must be carefully addressed. In this section, we discuss the opportunities and glioma-specific limitations of in vivo CAR-based immune cell engineering approaches for malignant glioma. Many of the strategies discussed remain conceptual or preclinical in this setting and have not yet been clinically validated. Although these approaches may help address key barriers to CAR-based immune cell therapies, further experimental and clinical studies will be required to determine their feasibility, safety, and therapeutic utility.

Malignant glioma, particularly GBM, is associated with a reduced pool of functional T cells in patients [13,14]. Although this challenge also applies to ex vivo engineered CAR-T cell therapies, it is particularly critical for CAR-based immune cell therapies that rely on patient-derived immune cells. However, the off-the-shelf nature of in vivo engineering approaches enables a substantial reduction in the time required to generate CAR-T cells, thereby providing greater flexibility in treatment strategies. For example, this approach could allow early postoperative adjuvant therapy before the initiation of chemoradiation, or even neoadjuvant therapy prior to surgery, in which T cells are likely to be in a better functional state than those after chemoradiation or at recurrence. Such treatment strategies, which are difficult to implement with ex vivo engineering approaches, may represent a significant advantage of in vivo CAR-T cell approaches.

To address the profound immunosuppressive microenvironment and antigenic heterogeneity of malignant glioma, in vivo-engineered CAR-T cell approaches may benefit from flexible dosing and targeting strategies. In particular, transient-expression platforms based on LNPs or polymeric delivery systems may require repeated administration to maintain therapeutic activity [99]. Beyond sustaining CAR expression, repeated dosing may also provide a continuous supply of newly generated CAR-T cells, which could potentially help counteract functional suppression within the tumor microenvironment. In contrast, repeated administration may be less feasible with viral vector-based approaches due to immune responses directed against the vector itself. Nevertheless, the ability to generate CAR-T cells in vivo without the need for repeated lymphodepletion may provide practical advantages for systemic re-dosing compared with conventional ex vivo CAR-T cell therapies.

The flexibility of in vivo engineering platforms may also facilitate adaptation to tumor evolution and antigenic heterogeneity. For example, vector redesign according to antigen profiles at recurrence, as well as pooled LNP formulations delivering distinct CAR constructs, may enable multi-targeting strategies to mitigate antigen escape and address heterogeneous antigen expression. Furthermore, the optimal target antigen and mode of CAR expression may vary among glioma-associated antigens depending on tumor specificity, expression heterogeneity, antigen escape potential, and normal tissue expression (Table 4). In this setting, the ability of in vivo CAR-T cell engineering platforms to support either transient or stable CAR expression may facilitate the development of antigen-tailored therapeutic strategies while improving the balance between efficacy and safety.

Beyond antigen heterogeneity, future in vivo CAR-based immune cell therapies for malignant glioma may also need to address spatially organized invasive tumor ecosystems. In addition, glioma stem cell populations maintained by developmental signaling pathways, including Notch and its extensive signaling crosstalk, may contribute to cellular plasticity, therapeutic resistance, and antigenic diversification, thereby limiting the durability of CAR-T cell responses [116]. These observations suggest that therapeutic strategies targeting glioma stem cell-associated antigens may help address an additional layer of resistance beyond conventional tumor-associated antigens.

Beyond stem cell-associated mechanisms of therapeutic resistance, combination strategies targeting mesenchymal transition, extracellular matrix remodeling, or invasion-associated niches may enhance the ability of CAR-engineered immune cells to control infiltrative and recurrent disease. In addition, emerging studies in solid tumors have demonstrated the feasibility of universal or multi-compartment targeting CAR strategies that simultaneously target tumor cells and stromal components, including cancer-associated fibroblasts (CAFs) [117,118]. Such approaches may help overcome both antigen heterogeneity and the immunosuppressive TME by disrupting stromal niches that support tumor progression, immune evasion, and T cell exclusion. Although malignant gliomas do not typically contain a classical CAF-rich stroma comparable to that in epithelial cancers, recent studies suggest that mesenchymal multicellular structures and extracellular matrix remodeling may play analogous roles within the glioma microenvironment [119]. Therefore, future in vivo CAR-based immune cell therapies may benefit from strategies targeting not only tumor-associated antigens but also stromal and mesenchymal components contributing to immune suppression and infiltrative tumor progression.

In vivo-engineered CAR-T cells, which reach brain tumors via systemic circulation similar to IV delivery, are likely to require design strategies that enhance their homing capacity to brain tumors. One potential approach involves chemokine receptors, such as C-X-C motif chemokine receptor (CXCR) 3, which have been identified as key mediators of T cell trafficking to the brain [120,121]. However, T cells targeted for in vivo engineering are largely within the naïve T cell compartment, where expression of these molecules is typically low. Notably, vectors engineered with anti-CD3 scFv to confer T cell tropism are known to induce T cell activation upon CD3 engagement, and thus activation-induced acquisition of trafficking capacity may potentially occur [122,123]. Supporting this concept, in a diffuse intrinsic pontine glioma mouse model, B7 homolog 3 (B7-H3)-targeting CAR-T cells engineered to express CXCR3-A, an isoform of CXCR3, have been reported to exhibit enhanced tumor trafficking and improved antitumor efficacy [124]. Similarly, CAR-T cells engineered to express CXCR1 or CXCR2, receptors for IL-8, have been developed to enhance trafficking to the tumor site by responding to IL-8 secreted by GBM and are currently being evaluated in clinical applications [125]. More broadly, chemokine-based strategies have also been explored in other solid tumors. For example, CAR-T cells engineered to express C-C motif chemokine receptor (CCR) 2, which binds CCL2, have been reported to exhibit enhanced migration and infiltration into mesothelin-expressing tumors and ovarian cancer [126,127].

In addition, CAR-macrophage approaches for GBM have recently attracted increasing attention [128,129]. The TME of GBM is characterized by abundant infiltration of myeloid cells [15,16,17]. Circulating myeloid cells exhibit superior trafficking and survival within the GBM tumor microenvironment. As precursors of macrophages, monocytes express high levels of chemokine receptors, including CCR2 and the colony-stimulating factor 1 receptor (CSF1R), which facilitate their recruitment to brain tumors that express ligands, such as CCL2 and CSF-1 [130,131]. Therefore, the concept of CAR-macrophages that leverage macrophages’ intrinsic properties may provide a trafficking advantage over CAR-T cells [132].

Early CD3ζ-based CAR macrophages exhibit antigen-specific phagocytic activity. However, their in vivo efficacy remains inferior to that of CAR-T cells [133]. Consequently, efforts are underway to develop CAR macrophages with optimized intracellular domains. Indeed, as described above, clinical trials of in vivo-engineered CAR-myeloid cell therapy targeting epithelial cancers have already been initiated, leveraging both the preferential uptake of LNPs by macrophages and their inherent capacity to infiltrate solid tumors.

Beyond CAR-macrophage approaches, CAR-NK cell therapies have also emerged as promising alternatives to CAR-T cell therapies for malignant glioma [134,135]. NK cells possess intrinsic antitumor activity through MHC-independent recognition mechanisms and may therefore retain activity against tumor cells that have undergone antigen loss or MHC downregulation. These properties may be particularly advantageous in malignant glioma, where marked antigenic heterogeneity and antigen escape represent major barriers to effective CAR-based immune cell therapies. In addition, CAR-NK therapies have demonstrated favorable safety profiles in early clinical studies and may be associated with reduced risks of severe CRS and neurotoxicity compared with CAR-T cell therapies. However, limited persistence, insufficient expansion, and the highly immunosuppressive glioma microenvironment remain important challenges that may restrict their therapeutic efficacy [136].

Interest in alternative immune cell engineering strategies is further reflected by the development of emerging in vivo CAR-based immune cell platforms designed to simultaneously generate both CAR-T and CAR-NK cells in vivo [95]. Such approaches may combine the long-term persistence and adaptive immune functions of T cells with the innate antitumor activity and resistance to antigen escape characteristic of NK cells. Although clinical experience remains limited, CAR-NK cell therapies and multi-lineage CAR engineering approaches may represent promising complementary strategies for overcoming antigen heterogeneity and other barriers associated with malignant glioma.

While current in vivo CAR engineering platforms primarily rely on systemic administration to modify circulating immune cells, alternative locoregional delivery strategies may be particularly attractive for malignant glioma. Local administration of nanoparticles or biomaterial-based scaffolds into the tumor or resection cavity could potentially improve vector retention while reducing off-target exposure. Such approaches may also facilitate the engineering of tumor-infiltrating immune populations, including macrophages and other myeloid cells, which constitute major cellular components of the glioma microenvironment. In particular, biomaterial-based scaffolds implanted within the tumor resection cavity may serve as localized platforms for in vivo CAR-based immune cell engineering, enabling the generation and programming of CAR-engineered immune cells directly at the tumor site. By reducing dependence on long-distance trafficking across the blood–brain barrier, these strategies may enhance local immune cell accumulation, persistence, trafficking to residual tumor cells, and overall antitumor activity.

Low-intensity pulsed ultrasound (LIPU) is a technique developed to transiently open the BBB and improve drug delivery. LIPU, in combination with IV-administered microbubbles, can transiently and reversibly open the BBB. Acoustic oscillation of microbubbles induced by ultrasound generates mechanical forces on the cerebral endothelium, temporarily disrupting tight junctions and increasing vascular permeability, thereby enhancing drug delivery to the brain. In a GBM mouse model, the combination of LIPU and CAR-T cell therapy has been reported to enhance CAR-T cell trafficking to CNS tumor sites and significantly prolong median survival [137].

Collectively, these challenges suggest that the limitations in developing in vivo-engineered CAR-T cell therapies for brain tumors are not attributable to a single barrier, but rather arise from the convergence of multiple factors, including intrinsic T cell properties and restricted trafficking across the BBB. Addressing these limitations will likely require integrated strategies that combine optimized vector design, enhancement of trafficking capacity, alternative immune cell platforms such as CAR-macrophages, and approaches to transiently modulate the BBB. A comprehensive understanding of these barriers and their interplay will be essential for the rational development of in vivo CAR-based immune cell therapies for brain tumors.

## 11. Potential Combination Strategies for In Vivo-Engineered CAR-T Cell Therapy in Malignant Glioma

Despite recent advances in CAR-T cell therapy, malignant glioma remains highly resistant because of its immunosuppressive microenvironment, limited CNS trafficking, and antigenic heterogeneity. Therefore, combination strategies will likely be required to improve therapeutic efficacy, and in vivo-engineered CAR-T cell platforms may provide unique opportunities for such approaches.

Among these strategies, combination with immune checkpoint inhibitors (ICIs) is a particularly attractive approach given the frequent expression of inhibitory immune checkpoint molecules, such as programmed death-ligand 1 (PD-L1), in GBM. Previous studies evaluating ex vivo engineered CAR-T cells in solid tumors, including GBM, have demonstrated that combination therapy with PD-1 blockade is generally feasible and tolerable. However, despite generally acceptable safety profiles, clinical efficacy has remained limited, with restricted CAR-T cell expansion and persistence observed in several studies [101]. These findings suggest that PD-1 inhibition alone may be insufficient to fully overcome the multiple immunosuppressive mechanisms operating within the glioma microenvironment, including myeloid-cell-mediated suppression, antigen heterogeneity, and limited T-cell trafficking into CNS tumors. Nevertheless, recent studies have suggested potential benefits of neoadjuvant immune checkpoint blockade in GBM, highlighting the importance of treatment timing and immune priming [138,139]. In this context, the improved accessibility and repeat-dose feasibility of in vivo-engineered CAR-T cell platforms may provide unique opportunities for more flexible and adaptive combination strategies with ICIs, including neoadjuvant approaches.

Oncolytic viruses may also serve as valuable combination partners for in vivo-engineered CAR-T cell therapy in malignant glioma. In addition to directly inducing tumor cell lysis, oncolytic viruses can promote local inflammation, antigen release, and activation of endogenous antitumor immunity within the glioma microenvironment [140]. Viral infection may further enhance the expression of inflammatory cytokines and chemokines, thereby promoting immune cell recruitment and facilitating CAR-T cell trafficking and activation within CNS tumors [141,142]. Such effects may be particularly important in GBM, which is characterized by a profoundly immunosuppressive microenvironment and limited baseline T-cell infiltration. Previous studies evaluating ex vivo engineered CAR-T cells have suggested that oncolytic viruses may enhance CAR-T cell efficacy by promoting antigen spreading and remodeling the tumor microenvironment into a more immune-reactive state. Therefore, integration of oncolytic virotherapy with in vivo-engineered CAR-T cell platforms may represent a promising strategy to improve CAR-T cell trafficking and function within CNS tumors.

Immune-stimulatory and cytotoxic dual-vector gene therapy approaches, such as HSV1 thymidine kinase (HSV1-TK) and Fms-like tyrosine kinase 3 ligand (Flt3L), may also complement in vivo-engineered CAR-T cell therapy for malignant glioma. HSV1-TK-mediated tumor cell death promotes antigen release, whereas Flt3L enhances dendritic-cell recruitment and activation within the tumor microenvironment [143,144]. Together, these effects may facilitate endogenous immune activation and create a more permissive microenvironment for in vivo-engineered CAR-T cells.

**Figure 3:**
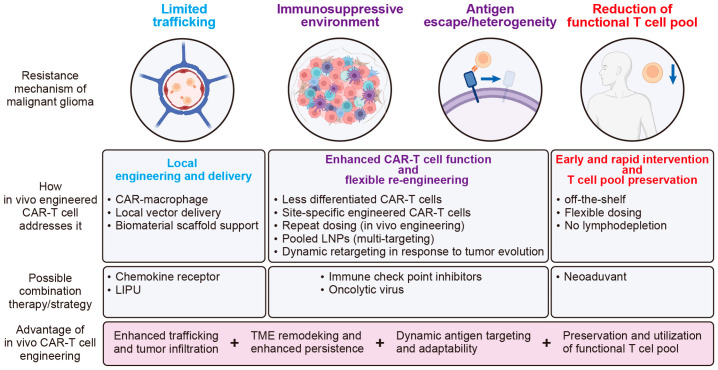
Schematic overview of how in vivo-engineered chimeric antigen receptor (CAR)-T cell therapies may overcome major resistance mechanisms in malignant glioma. Potential advantages include improved trafficking and tumor infiltration, tumor microenvironment (TME) remodeling, dynamic adaptation to antigenic evolution through repeat dosing and re-engineering strategies, and preservation of functional T cell populations. Combination strategies such as chemokine receptor engineering, Low-intensity pulsed ultrasound (LIPU), immune checkpoint blockade, and oncolytic virotherapy may further enhance therapeutic efficacy. LNP, Lipid nanoparticle. The schematic was created using BioRender.com.

Collectively, these combination approaches may represent promising strategies for overcoming the complex resistance mechanisms that limit the efficacy of immunotherapy in malignant glioma (Figure 3).

## 12. Conclusions

Nearly a decade has passed since the first CAR-T cell therapy was approved by the FDA. During this time, remarkable clinical successes have been achieved, particularly in hematologic malignancies, while key limitations of current ex vivo engineering approaches have also become increasingly apparent. In parallel, advances in viral vector engineering and nanotechnology have enabled the clinical translation of in vivo gene therapy, positioning in vivo CAR-T cell engineering as a promising new therapeutic paradigm to overcome these challenges in immunotherapy. Early clinical data on in vivo-engineered CAR-T cell therapies for hematologic malignancies are emerging, and their application to solid tumors is also underway. However, developing this approach for malignant glioma will require not only leveraging the logistical and biological advantages of in vivo CAR-T cell engineering but also a precise understanding of the unique challenges of treating malignant glioma and the implementation of strategies to address them. Potentially promising approaches may include optimizing CAR design, such as CXCR3-engineered CAR-T cells to enhance tumor trafficking; combination strategies integrating LIPU with CAR-T or CAR-macrophage therapies to improve immune activation and CNS delivery; and optimized treatment strategies, such as perioperative or early post-surgical administration, to maximize therapeutic efficacy in malignant glioma.

## Figures and Tables

**Table 1:** Comparison of ex vivo and in vivo CAR-T cell engineering approaches.

Feature	Ex Vivo CAR-T Cell Engineering	In Vivo CAR-T Cell Engineering
Manufacturing	Patient-specific cell collection, engineering, expansion, and reinfusion	Direct genetic engineering of endogenous immune cells
Time to treatment	Several weeks	Potentially same-day administration
Manufacturing cost	High (>USD 500,000 per product; >USD F1–1.5 million total treatment cost)	Potentially lower; cost reduction remains theoretical and requires clinical validation
Manufacturing scalability	Limited by individualized manufacturing	Potentially scalable and off-the-shelf
CAR expression	Typically stable genomic integration	Transient, stable, or site-specific depending on platform
Lymphodepletion	Usually required	Platform-dependent; may be reduced or omitted
Immune system integrity	Lymphodepletion may impair endogenous immunity	Potentially better preservation of endogenous immune responses
T cell fitness	May be impaired by prolonged ex vivo manipulation and expansion	Potentially preserved through engineering in the native physiological environment
Safety concerns	Lymphodepletion- and treatment-related toxicities	Vector/delivery-related toxicities, off-target transduction, and immune-mediated toxicities
Clinical readiness	Clinically established; multiple FDA-approved products	Early clinical development; no approved products

**Table 2:** Clinical trials of lentiviral vector-mediated in vivo-engineered CAR-based immune cells for cancers.

Clinical Trial Number	Phase	Product Name	Disease	Target Antigen	Target Cell	Vector	Injection Route	Number of Dosing	Expression Duration	Company	Source Type
NCT07075185	Phase 1	KLN-1010	R/R multiple myeloma	BCMA	T cells	LV	IV	Single	Stable	Kelonia Therapeutics	ClinicalTrials.gov Company disclosure Conference abstract [89]
NCT06743503	Phase 1	UB-VV400	R/R aggressive lymphoma	CD22	T cells	LV	IV	Single	Stable	Umoja Biopharma	ClinicalTrials.gov Company disclosure
NCT06528301	Phase 1	UB-VV111	R/R CD19^+^ B cell Malignancies	CD19	T cells	LV	IV	Single	Stable	Umoja Biopharma	ClinicalTrials.gov Company disclosure
NCT07284927, NCT07312630	Phase 1	LV009	R/R CD19^+^ hematolymphoid malignancies	CD19	T cells	LV	IV	Single	Stable	PersonGen BioTherapeutics	ClinicalTrials.gov Company disclosure
NCT07002112	Phase 1	LB2501	R/R B cell malignancies	CD19 and CD20 (dual target)	T cell	LV	IV	Single	Stable	Legend Biotech	ClinicalTrials.gov Company disclosure
NCT06691685, NCT06791681	Early Phase 1	ESO-T01	R/R multiple myeloma	BCMA	T cells	LV	IV	Single	Stable	Esobiotech	ClinicalTrials.gov Company disclosure Peer review paper [90]
NCT07376642	Early Phase 1	IMV101	R/R B cell non-Hodgkin lymphoma	CD19	T cells	LV	IV	Single	Stable	Shenzhen Immunofoco Biotechnology	ClinicalTrials.gov Company disclosure Conference abstract [91]
NCT07395479	Early Phase 1	V001	Advanced malignant tumors (including hematological malignancies and solid tumors)	BCMA, GPRC5D, DLL3 and FcRH5 (by cohort)	T cells	LV	IV	Single	Stable	Undisclosed	ClinicalTrials.gov Company disclosure
NCT07101705	Early Phase 1	OriV508	R/R B cell hematological malignancies	BCMA and CD19 (dual target)	T cells	LV	IV	Single	Stable	OriCell Therapeutics	ClinicalTrials.gov Company disclosure
NCT07065279, NCT06689917, NCT06678282, NCT06890065, NCT06940960, NCT06514768	Not applicable	JY231	R/R B cell Malignancies	CD19	T cells	LV	IV	Single	Stable	Shenzhen Genocury Biotech	ClinicalTrials.gov Company disclosure
NCT07336823	Early Phase 1	JY232	R/R multiple myeloma	BCMA	T cells	LV	IV	Single	Stable	Shenzhen Genocury Biotech	ClinicalTrials.gov Company disclosure
NCT06539338	Phase 1	INT2104	R/R B cell Malignancies	CD20	T cells and NK cells	LV	IV	Single	Stable	Kite Pharma	ClinicalTrials.gov Company disclosure

The primary endpoints of all studies were safety and tolerability. All trials were recruiting at the time of data collection, except NCT06539338 (active, not recruiting). Undisclosed indicates that the relevant information has not been publicly disclosed and remains proprietary to the sponsoring company. R/R, refractory/relapsed; LV, lentivirus; IV, intravenous; BCMA, B cell maturation antigen; GPRC5D, G protein-coupled receptor class C group 5 member D; DLL3, Delta-like ligand 3; FcRH5, Fc receptor homolog 5; NK, natural killer.

**Table 3:** Clinical trials of non-lentiviral vector-mediated in vivo-engineered CAR-based immune cells for cancer.

Clinical Trial Number	Phase	Product Name	Disease	Target Antigen	Target Cell	Vector	Injection Route	Number of Dosing	Expression Duration	Company	Source Type
NCT06618313	Not applicable	JCXH-213	R/R B cell non-Hodgkin lymphoma	CD19	T cells	LNP	IV	Not specified	Transient	Immorna Biotechnology	ClinicalTrials.gov Company disclosure
NCT05969041	Phase 1	MT-302	Metastatic or advanced epithelial cancer	TROP2	Myeloid cells	LNP	IV	Multiple	Transient	Create Medicines	ClinicalTrials.gov Company disclosure Conference abstract [92]
NCT06478693	Phase 1	MT-303	Advanced or metastatic GPC3-expressing cancers	GPC3	Myeloid cells	LNP	IV	Multiple	Transient	Create Medicines	ClinicalTrials.gov Company disclosure Conference abstract [93]
NCT07334119	Phase 1	MT-304	Advanced HER2-expression solid tumors	HER2	NK cells and myeloid cells	LNP	IV	Multiple	Transient	Create Medicines	ClinicalTrials.gov Company disclosure
NCT07398963	Phase 1	RGV005	R/R B cell lymphoma	CD19	T cells and myeloid cells	OVV	IV or IT	Single	Transient	Undisclosed	ClinicalTrials.gov
NCT07294625	Early Phase 1	LVIVO-TaVec200	R/R multiple myeloma	GPRC5D	T cells	Undisclosed	IV	Single	Undisclosed	Legend Biotech	ClinicalTrials.gov Company disclosure
NCT07239323	Phase 1	Undisclosed	R/R hematological malignancies	CD19	T cells	Undisclosed	IV	Single	Undisclosed	Chongqing Precision Biotech	ClinicalTrials.gov Company disclosure

The primary endpoints of all studies were safety and tolerability. All trials were recruiting at the time of data collection. Undisclosed indicates that the relevant information has not been publicly disclosed and remains proprietary to the developing company. R/R, refractory/relapsed; LNP, lipid nanoparticle; IV, intravenous; TROP2, Trophoblast cell surface antigen 2; GPC3, Glypican-3; HER2, Human epidermal growth factor receptor 2; NK, natural killer; OVV, oncolytic vaccinia virus; IT, intratumoral; GPRC5D, G protein-coupled receptor class C group 5 member D.

**Table 4:** Comparison of representative malignant glioma-associated antigens for in vivo CAR-T cell engineering.

Antigen	Prevalence	Heterogeneity	On-Target/Off-Tumor Toxicity	Clinical Ex Vivo CAR-T Cell Experience	Preferred Expression Mode	Refs.
EGFRvIII	20–30% in GBM	High	Minimal	NCT01454596 [100], NCT02209376 [20], NCT03726515 [101], NCT05660369 [23]	Stable	[102]
IL13Rα2	50–60% in GBM	High	Low	NCT00730613 [103], NCT01082926, NCT02208362 [9], NCT05168423 [22], NCT04003649	Transient or stable	[104,105]
B7-H3	~75% in GBM > 90% in DIPG	Low-Moderate	Moderate	NCT04185038 [106], NCT05835687, NCT07390539, NCT05474378	Transient	[107,108]
HER2	~80% in GBM	Moderate	Moderate-High	NCT01109095 [109], NCT03500991 [110]	Transient	[111]
EphA2	90% In GBM	Moderate-High	High	NCT03423992 [112]	Transient	[113]
GD2	~40% in GBM Highly expressed in DIPG	Moderate	Moderate-High	NCT03170141 [114], NCT04196413 [10], NCT04099797 [115]	Transient	[10,114]

## Data Availability

No new data were created or analyzed in this study.

## Abbreviations

The following abbreviations are used in this manuscript:

CAR	Chimeric antigen receptor
scFv	Single-chain variable fragment
NK	Natural killer
GBM	Glioblastoma
IL13Rα2	Interleukin-13 receptor alpha 2
EGFR	Epidermal growth factor receptor
ICANS	Immune Effector Cell-Associated Neurotoxicity Syndrome
DIPG	Diffuse intrinsic pontine glioma
IV	Intravenous
ICV	Intracerebroventricular
TIAN	Tumor inflammation-associated neurotoxicity
CRS	Cytokine release syndrome
BBB	Blood–brain barrier
TME	Tumor microenvironment
TMZ	Temozolomide
CTLA-4	Cytotoxic T-lymphocyte-associated protein 4
MDSC	Myeloid-derived suppressor cell
EGFRvIII	Epidermal growth factor receptor variant III
COL1A1	Collagen type 1 alpha chain
CNS	Central nervous system
FDA	Food and Drug Administration
LV	Lentivirus
IL	Interleukin
TCR	T cell receptor
MHC	Major histocompatibility complex
iPSC	Induced pluripotent stem cells
GVHD	Graft-versus-host disease
LNP	Lipid nanoparticle
AAV	Adeno-associated virus
EMA	European Medicine Agency
TRAC	T cell receptor alpha constant
PD-1	Programmed cell death protein 1
AAVS	Adeno-associated virus integration site 1
EDV	Enveloped delivery vehicle
GMP	Good manufacturing practice
DARPin	Designed ankyrin repeat protein
MDF	Multidomain fusion
PEI	Polyethylenimine
PBAE	Poly (β-amino ester)
PEG	Poly(ethylene glycol)
CCL	C-C motif chemokine ligand
EV	Extracellular vesicles
VLP	Virus-like particles
VFMN	Virus mimetic fusogenic nanoparticle
BCMA	B-cell maturation antigen
AST	Aspartate Aminotransferase
TNF	Tumor necrosis factor
VSV-G	Vesicular stomatitis virus glycoprotein
PR	Partial response
VGPR	Very good partial response
TROP2	Trophoblast cell surface antigen 2
LDL-R	Low-density lipoprotein receptor
GPC3	Glypican-3
HER2	Human epidermal growth factor receptor 2
CAF	Cancer-associated fibroblast
CXCR	C-X-C motif chemokine receptor
B7-H3	B7 homolog 3
CCR	C-C motif chemokine receptor
CSF1R	Colony-stimulating factor 1 receptor
LIPU	Low-intensity pulsed ultrasound
ICI	Immune checkpoint inhibitor
PD-L1	Programmed death-ligand 1
HSV1-TK	Herpes simplex virus type 1 thymidine kinase
Flt3L	Fms-like tyrosine kinase 3 ligand
DAMP	Damage-associated molecular pattern
